# High Specific Selectivity and Membrane-Active Mechanism of Synthetic Cationic Hybrid Antimicrobial Peptides Based on the Peptide FV7

**DOI:** 10.3390/ijms18020339

**Published:** 2017-02-06

**Authors:** Tingting Tan, Di Wu, Weizhong Li, Xin Zheng, Weifen Li, Anshan Shan

**Affiliations:** 1 Institute of Animal Nutrition, Northeast Agricultural University, Harbin 150030, China; 15684244286@163.com (T.T.); woodyneau@163.com (D.W.); laowu2003@wfu.edu.cn (W.L.); 2 College of Animal Science and Technology, Jilin Agricultural University, Changchun 130118, China; zhengxinjilin@126.com; 3 Institute of Animal Nutrition and Feed Science, College of Animal Science, Zhejiang University, Hangzhou 310058, China; wfli@zju.edu.cn

**Keywords:** microbiology, antimicrobial peptide, amino acid, FV7, biofilm

## Abstract

Hybrid peptides integrating different functional domains of peptides have many advantages, such as remarkable antimicrobial activity, lower hemolysis and ideal cell selectivity, compared with natural antimicrobial peptides. FV7 (FRIRVRV-NH_2_), a consensus amphiphilic sequence was identified as being analogous to host defense peptides. In this study, we designed a series of hybrid peptides FV7-LL-37 (17–29) (FV-LL), FV7-magainin 2 (9–21) (FV-MA) and FV7-cecropin A (1–8) (FV-CE) by combining the FV7 sequence with the small functional sequences LL-37 (17–29) (LL), magainin 2 (9–21) (MA) and cecropin A (1–8) (CE) which all come from well-described natural peptides. The results demonstrated that the synthetic hybrid peptides, in particular FV-LL, had potent antibacterial activities over a wide range of Gram-negative and Gram-positive bacteria with lower hemolytic activity than other peptides. Furthermore, fluorescent spectroscopy indicated that the hybrid peptide FV-LL exhibited marked membrane destruction by inducing outer and inner bacterial membrane permeabilization, while scanning electron microscopy (SEM) and transmission electron microscopy (TEM) demonstrated that FV-LL damaged membrane integrity by disrupting the bacterial membrane. Inhibiting biofilm formation assays also showed that FV-LL had similar anti-biofilm activity compared with the functional peptide sequence FV7. Synthetic cationic hybrid peptides based on FV7 could provide new models for combining different functional domains and demonstrate effective avenues to screen for novel antimicrobial agents.

## 1. Introduction

Naturally occurring antimicrobial peptides (AMPs) are considered potential anti-infective agents primarily due to their antimicrobial or immunomodulatory properties [1,2,3,4,5,6,7,8,9]. AMPs are 12 to 50 amino acids in length and are amphiphilic, containing 2 to 9 basic residues (R or K) and >50% hydrophobic residues [1,5]. Thousands of AMPs that demonstrate potent antimicrobial activities are rapidly mobilized to neutralize a broad range of microbes, including viruses, bacteria, protozoa and fungi, isolated from comprehensive species such as sheep, cow, pig and even humans [10,11,12]. As potent weapons against infectious microbes, including multidrug-resistant bacteria, AMPs have been regarded as a new class of therapeutic drugs which could alter antibiotics due to their broad spectrum of antibacterial activity and overwhelming superiority with respect to bacterial drug resistance. However, several concerns may weaken their development as antimicrobial agents ultimately, which include their potential cytotoxicity, poor antimicrobial activity based on peptide concentration and weak physiological stability [13].

**Figure 1:**
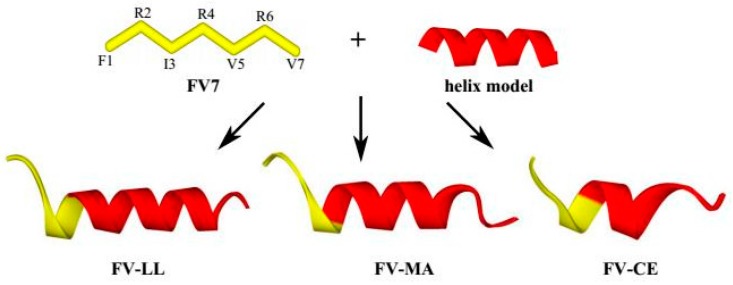
Three-dimensional structure projections of peptides and the process of hybrid peptide formation. FV7 is yellow, the helix model of the core sequences from native peptide (LL37, magainin 2 and cecropin A) is red.

Various modifications such as substitutions of specific amino acids [14] and truncation of parental peptides [15] have been employed to improve antimicrobial activity and physiological stability and reduce cytotoxic effects simultaneously. However, these changes were short of the systematic scientific rationale which aimed to improve AMP design principles. The synthesis of hybrid peptides, which is a simple effective strategy to design novel AMPs, combined the advantages of different native peptides. These hybrid peptides show great potential at interfaces in view of their inherent amphiphilic character and ability to readily aggregate in bulk solution [16]. Hybrid peptides with increased antimicrobial activity compared to the parent peptides have been constructed by other researchers, with improvements thought to improve their secondary structure [17,18]. As previously reported, several peptides integrating the FV7 (FRIRVRV-NH_2_) sequences (function sequences of inhibiting biofilm) have been tested positively for antimicrobial activity. Therefore, the FV7 sequence can serve as a basis for the iterative design of improved peptides [19,20]. It was also reported that LL-37 (17–29) (LL), which is the shortest antimicrobial and anticancer peptide derived from human cathelicidin identified to date that includes an amphiphilic helix rich in positively charged side chains, effectively competed with anionic phosphatidylglycerols in bacterial membranes and displayed a selective toxic effect on bacteria but not human cells [21]. The small effective peptide between residues 9 and 21 of the amphiphilic, antibiotic peptide magainin 2 (MA) demonstrated a stable amphiphilic α-helix which was believed to be an important structural feature for membrane-permeating peptides [22]. Cecropin A (1–8) (CE) has been frequently used to synthesize novel hybrid peptides with other native peptide sequences [23]. The antibacterial activity of the CM11 peptide (WKLFKKILKVL-NH_2_), a short cecropin-melittin hybrid peptide, was studied against antibiotic-resistant strains. In particular, the 11-residue sequence WKLFKKILKVL-NH_2_ (Pep3), which is derived from the well-known cecropin A (1–7)-melittin (2–9) hybrid peptide [24,25,26], is sufficient for antifungal and antibacterial activities [27,28]. Considering the advantages of the above peptide sequences, we designed a series of hybrid peptides by combining the FV7 sequence with the small functional LL-37, magainin 2 and cecropin A sequences, which demonstrated that hybridization is an effective way to optimize native peptides (Figure 1). On the basis of the cathelicidin family, these hybrid peptides share a highly conserved N-terminal “cathelin” domain and a highly variable C-terminal antimicrobial region. Therefore, there is a strong focus on developing novel hybrid peptides of therapeutic value based on the peptide FV7.

We surmised that these modifications would conserve the lower hemolytic activity and higher broad spectrum antibacterial activity while increasing antibacterial activity and stability. Antibacterial activities were examined to measure against bacterial organisms ranging from Gram-positive, such as *Staphylococcus aureus* (*S. aureus*), *Bacillus subtilis* (*B. subtilis*) and *Staphylococcus epidermidis* (*S. epidermidis*), to Gram-negative bacteria, such as *Escherichia coli* (*E. coli*), *Pseudomonas aeruginosa* (*P. aeruginosa*) and *Salmonella typhimurium* (*S. typhimurium*) by a minimum inhibitory concentration (MIC). The secondary structures of hybrid peptides were determined by circular dichroism (CD) in membrane-mimicking environments which include 30 mM sodium dodecyl sulfate (SDS) and 50% trifluoroethyl alcohol (TFE). The potential cytotoxic effects against mammalian cells were determined based on human red blood cells (hRBC) hemolysis, and their stability was investigated by assessing the antibacterial activities of the peptides in the presence of cations and heat. The outer membrane permeability, inner membrane permeability, scanning electron microscopy (SEM), and transmission electron microscopy (TEM) were employed to investigate potential membrane destruction mechanisms.

## 2. Results

### 2.1. Peptide Design and Characterization

Matrix-assisted laser desorption/ionization time-of-flight mass spectroscopy (MALDI-TOF MS), reverse-phase high-performance liquid chromatography (RP-HPLC) and electrospray ionization-mass spectrometry (ESI-MS) were applied to verify the molecular weight, purity (>95%) and identity of the peptides, respectively. Table 1 summarized the theoretically calculated and measured molecular weights of the peptides. As Table 1 showed, the measured molecular weight value was consistent with the calculated value, suggesting that the peptides were successfully synthesized. All the designed hybrid peptides presented cationic activity (>+4) and varied hydrophobicities (>50%). The obvious hydrophobic order indicating the hydrophobicity of the hybrid peptides in aqueous solution was FV7 > FV-MA > FV-CE > FV-LL. All the positively charged residues and hydrophobic residues of these peptides were more dispersed, indicating a more idealized amphiphilicity. The positively charged FV-LL (+7), FV-MA (+5) and FV-CE (+7) were further increased compared with those parental peptides. The relative hydrophobic moments (μHrels) of all the peptides are similar to one another, at approximately 0.46–0.57.

### 2.2. The Hybrid Peptides Have Typical α-Helix Structures

**Figure 2:**
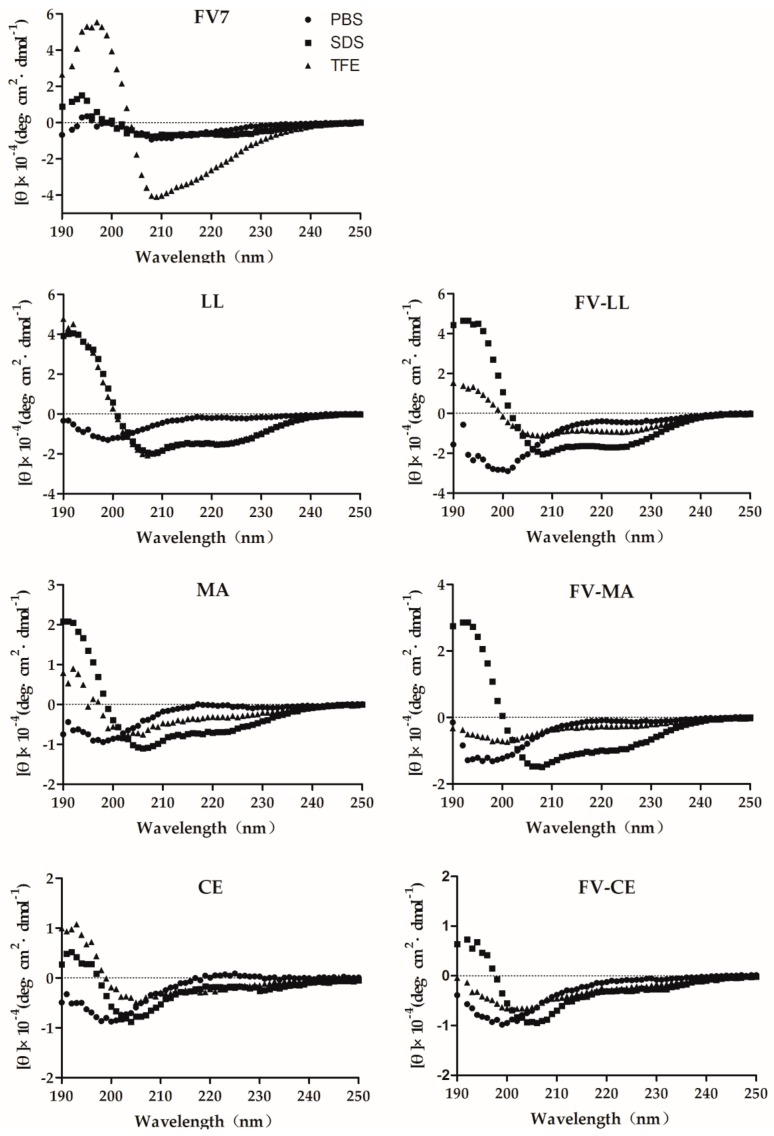
The circular dichroism (CD) spectra of the peptides in 10 mM phosphate-buffered saline (PBS; pH 7.4) (●), 30 mM sodium dodecyl sulfate (SDS) (▪) and 50% trifluoroethyl alcohol (TFE) (▲). The peptide concentration was fixed at 150 μM.

Circular dichroism (CD) spectroscopy of FV7, LL, MA, CE and all the hybrid peptides was performed at 150 μM in 10 mM phosphate-buffered saline (PBS), 30 mM sodium dodecyl sulfate (SDS) and 50% trifluoroethyl alcohol (TFE) (Figure 2). The CD spectra of parental peptide FV7 significantly demonstrated a β-sheet in the SDS micelles, with a maximum near 200 nm and a minimum just below 220 nm [29]. All of the hybrid peptides displayed an unordered conformation in phosphate buffer, which presented a strong minimum peak near 200 nm. When 30 mM of SDS and 50% TFE were mixed with FV-LL and FV-MA, an increase in the mean residue ellipticity was observed at 208 and 225 nm, which is consistent with the formation of an α-helix [30], which has structures similar to LL and MA. The conformation of FV-CE was the same in the presence of SDS or TFE as in buffer, suggesting a random coil structure. The structure of the two hybrid fragments is close to that of the latter fragments (LL, MA, CE).

### 2.3. FV-LL Is the Optimal Hybrid Peptide Compared with the Parental Peptides and Other Peptides in the Antimicrobial Activities

The results for the antibacterial activities against Gram-negative and Gram-positive bacteria are summarized in Table 2. Consequently, the hybrid peptides FV-LL, FV-MA and FV-CE showed a marked ability to inhibit Gram-negative and Gram-positive bacteria compared with the parental peptides. In contrast with the peptide FV7, the hybrid peptide FV-LL displayed approximately 4–32-fold higher antibacterial activity across bacteria, while FV-MA displayed approximately 2–16-fold higher antibacterial activity. Of the designed hybrid peptides, FV-LL displayed approximately 2–8-fold higher antibacterial activity than FV-MA. Meanwhile FV-CE showed the similar effect to the activity of the control natural peptide melittin (ME26). These assays confirmed that these hybrid peptides showed an extended antibacterial spectrum in comparison to their parent peptide FV7. FV7 and LL had similar antibacterial activity, however this was unsatisfactory. However, the peptides MA and CE showed hardly any activity on bacteria including Gram-positive bacteria and Gram-negative bacteria. Their measured values of minimum inhibitory concentration were greater than 64 μM. The assays indicated that cationic hybrid peptides combining FV7 with several minimal effect fragments of the natural antibacterial peptides had been significantly improved with respect to antibacterial activity compared with the above peptides. The minimum inhibitory concentration (MIC) for both FV7 and LL was 16 μM against *E. coli* ATCC 25922, however the detected value of the synthetic hybrid peptide FV-LL had reached 1 μM, which displayed approximately 16-fold higher antibacterial activity.

### 2.4. The Hybrid Peptides, Particularly FV-LL, Had Lower Hemolytic Activity Than the Control Peptide Melittin (ME26)

**Figure 3:**
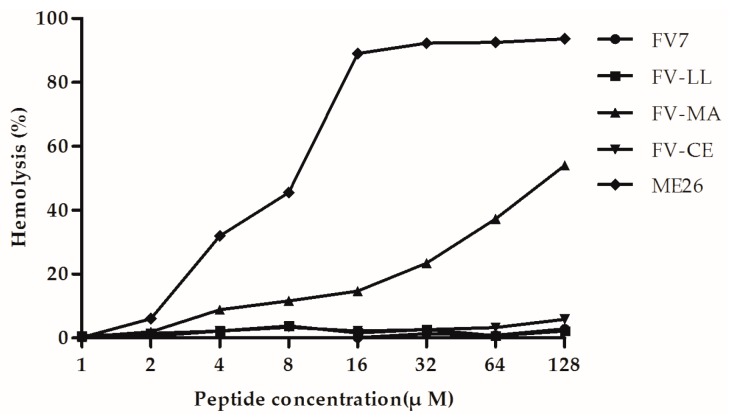
Hemolytic activities of the peptides against fresh human erythrocytes. The release of hemoglobin was monitored with a Microplate Autoreader (SpectraMax M5, Molecular Devices, San Francisco, CA, USA) by measuring the absorbance at 570 nm. Data are the averages of three independent experiments.

The hemolytic activity of the peptides against highly sensitive human erythrocytes was determined to assess the cytotoxicity against mammalian cells (Figure 3). Neither FV-LL nor FV-CE caused 5% hemolysis at the maximal concentration of 128 µM. In fact, neither of these peptides displayed any hemolytic activity even at the maximum concentration. The parental peptide FV7 showed 5% hemolysis at 4 µM and caused approximately 15% hemolysis at 128 µM. As a control peptide, ME26 caused 5% hemolysis at 0.25 µM and resulted in complete hemolysis at concentration greater than 4 µM. FV-MA demonstrated higher hemolytic activity at each concentration when compared to FV7.

### 2.5. FV-LL Was the Most Resistant of the Peptides in Stability Studies

The antibacterial activities of the peptides were tested using a sensitivity assay following the addition of physiological concentrations of different salts and heat (Table 3). The peptide FV7 retained activity against the Gram-negative bacterium *E. coli* ATCC 25922 in the presence of all of the cations except NH_4_^+^ and Fe^3+^ and almost lost activity against the Gram-positive bacterium *S. aureus* ATCC 29213. The hybrid peptides were almost tolerant to physiological salts, particularly FV-LL, for which results were basically similar to those of the control group.

### 2.6. The Hybrid Peptides Increased Outer and Inner Membrane Permeabilities

**Figure 4:**
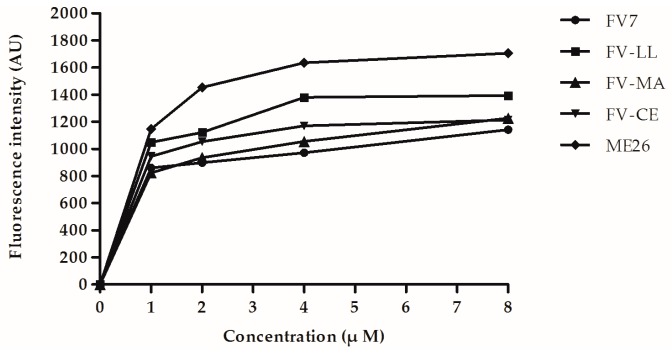
The outer membrane permeability of the parental and hybrid peptides.

To explore the mechanism of action of hybrid peptides, the penetration of the Gram-negative bacterial cell membrane was used for testing. The ability of peptides to permeabilize the outer membrane was evaluated by using the fluorescence-based *N*-phenyl-1-naphthylamine (NPN) uptake assay. Increased fluorescence was measured when NPN could traverse the destabilizing outer membrane and incorporate into the damaged membrane. As shown in Figure 4, all of the peptides were able to permeabilize the *E. coli* UB1005 outer membrane in a dose-dependent manner. All of the peptides could permeabilize the outer membrane at different concentrations ranging from 1 to 8 μM. At a lower concentration, such as 1 μM, FV-LL had similar permeabilization levels with respect to the control peptide ME26, which was much higher than hybrid peptides FV-MA and FV-CE. Compared with the parental peptide FV7, FV-LL had a remarkable ability to permeabilize the outer membrane at higher concentrations.

**Figure 5:**
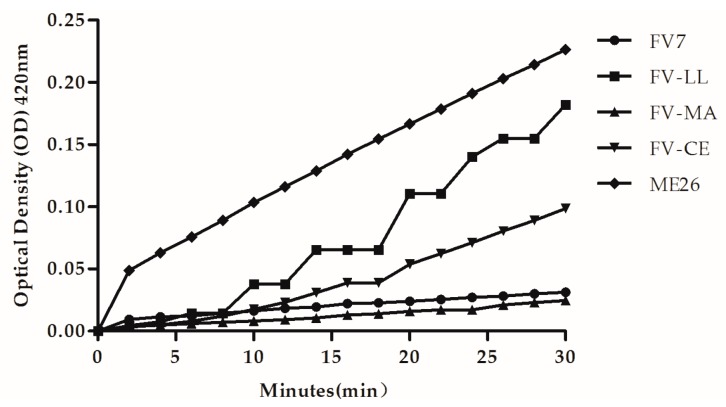
The inner membrane permeability of the peptides. The hydrolysis of *o*-Nitrophenyl-β-d-galactopyranoside (ONPG) due to release of cytoplasmic β-galactosidase of *E. coli* UB1005 treated by 1× MIC peptides was measured spectroscopically at absorbance of 420 nm as a function of time.

If the peptide induced permeabilization of the inner membrane, *o*-Nitrophenyl-β-d-galactopyranoside (ONPG) entered the cytoplasm and was degraded by β-galactosidase, producing *o*-nitrophenol that produced absorbance at 420 nm. As shown in Figure 5, at their 1× MIC, all of the peptides induced increases in the inner membrane permeability. However, the native peptide ME26 retained the highest ability for inner membrane permeability. All of the peptides had similar membrane permeability at two minutes except ME26. The obvious membrane permeability order is ME26 > FV-LL > FV-CE > FV7 > FV-MA. The peptide FV-LL was the most effective of all designed hybrid peptides. However, FV-MA induced slight inner membrane permeabilization within 30 min, which was similar to the parental peptide FV7.

### 2.7. All Hybrid Peptides Caused a Certain Degree of Membrane Damage in Scanning Electron Microscopy (SEM) and Transmission Electron Eicroscopy (TEM), Particularly FV-LL

**Figure 6:**
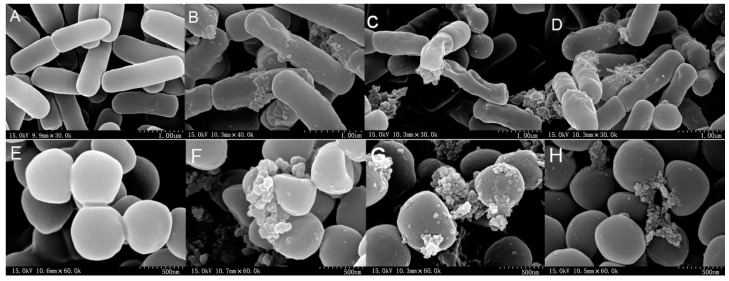
Scanning electron microscopy (SEM) micrographs of *E. coli* ATCC 25922 and *S. aureus* ATCC 29213 cells treated with peptides at their 1× MICs at about 1 h. *E. coli* ATCC 25922: (**A**) Control; (**B**) FV-LL; (**C**) FV-MA; (**D**) FV-CE. *S. aureus* ATCC 29213; (**E**) Control; (**F**) FV-LL; (**G**) FV-MA; (**H**) FV-CE. The control did not contain the peptides.

The membrane integrity and morphological changes of *E. coli* ATCC 25922 and *S. aureus* ATCC 29213 cells treated with hybrid peptides were studied by SEM. As is shown in Figure 6, the control with no peptides exhibited a bright and smooth surface without cellular debris.

However, in the presence of the peptides at 1× MIC, the treated bacteria appeared to clump with more crenated surfaces. Partial membrane atrophy, corrugation and damage were observed in the bacteria cells with the peptides. The membrane surfaces of the FV-LL-treated bacteria cells showed more roughening and blebbing, as evidenced by the formation of aggregates on the cell surface, compared with the effects observed in the FV-MA-and FV-CE-treated bacteria. *E. coli* ATCC 25922 and *S. aureus* ATCC 29213 cells produced significant membrane corrugation, blebbing and damage after treated with the hybrid peptides for 60 min.

**Figure 7:**
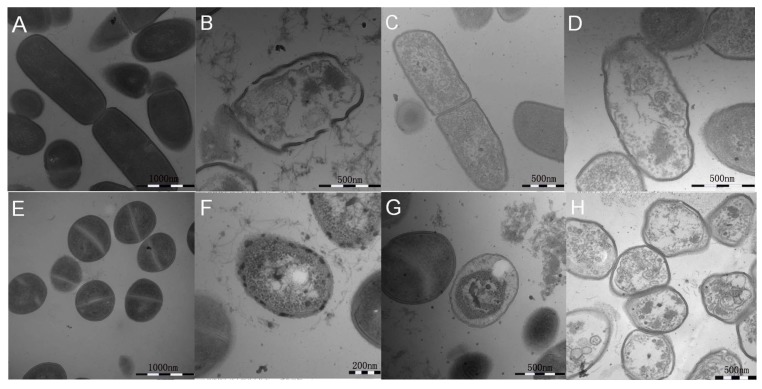
Transmission electron microscopy (TEM) micrographs of *E. coli* ATCC 25922 and *S. aureus* ATCC 29213 cells treated with the peptides at their 1× MICs at about 1 h. *E. coli* ATCC 25922: (**A**) Control; (**B**) FV-LL; (**C**) FV-MA; (**D**) FV-CE. *S. aureus* ATCC 29213: (**E**) Control; (**F**) FV-LL; (**G**) FV-MA; (**H**) FV-CE. The control did not contain the peptides.

To further study the antimicrobial mechanisms by observing ultrastructural changes of bacterial membrane integrity following peptide treatment in *E. coli* ATCC 25922 and *S. aureus* ATCC 29213, TEM studies were conducted. After treatment with hybrid peptides at their 1× MICs, the cell membrane was disrupted and visible pores with large diameters were observed. After 1 h, although the membrane integrity was adversely affected by treatment with FV-LL, FV-MA and FV-CE, the leakage of intracellular content could be observed. The treatment with hybrid peptides induced more rupture of membranes and release of intracellular contents compared to in the absence of peptide (Figure 7).

### 2.8. FV-LL Was More Effective in Inhibition of Biofilm Formation Compared with FV7

**Figure 8:**
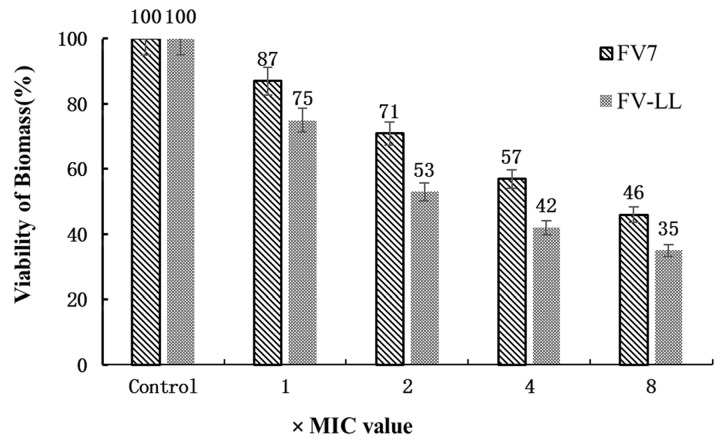
Anti-biofilm activity of the peptides. The viability of *P. aeruginosa* PAO1 biofilm was reduced after 24-h treatment at higher concentrations of FV7 and FV-LL. The test was performed at least four times in triplicate.

The anti-biofilm activity of the optimized hybrid peptide FV-LL and the parental peptide FV7 on *P. aeruginosa* PAO1 at different higher concentrations were investigated to prevent the formation of biofilm. From Figure 8, the optimized hybrid peptide FV-LL and parental peptide FV7 showed a dose-dependent inhibition of biofilm formation. After treating biofilms with peptides, the amount of survival biofilm was progressively reduced, with increasing concentrations (1–8× MIC) of FV7 and FV-LL. In each concentration, the ratio of FV-LL is lower than FV7, indicating that its inhibition of biofilm formation is better than that of FV7.

## 3. Discussion

The objective of the present study was to search for more effective peptides with a broader spectrum of antibacterial ability. Here we identified the highly potent synthetic hybrid peptides, despite the existence of natural antimicrobial peptides LL-37, magainins and cecropins. Therefore, several design strategies have been devised to develop novel peptides with higher antibacterial activity and lower cytotoxicity. For instance, the designed α-helical hybrid peptides based on natural peptides such as PRW4, fowlicidin-2, protegrin-3 and tritrpticin sequences to gain insights into their abilities (selectivities, physiological stabilities and endotoxin neutralization capabilities) exhibited high cell selectivity [13].

As previously reported, the consensus amphiphilic sequence FV7 (FRIRVRV-NH2) with three cationic residues and four hydrophobic amino acids have been tested positively for anti-biofilm activity, which was analogous to host defense peptides [19]. The synthetic hybrid peptide R-FV-I16 had potent antibacterial activity over a wide range of Gram-negative and Gram-positive bacteria, and had lower hemolytic activity and cytotoxicity. This embedded FV7 in the middle position of the RI16, replacing the functionally defective RR7 sequence [20]. In this study, a series of effective hybrid cationic antimicrobial peptides were designed that combined the parental peptide FV7 with functional sequence fragments (LL, MA and CE) from natural antibacterial peptides to generate AMPs with enhanced therapeutic potential. Three synthetic hybrid peptides were selected to be evaluated as AMPs. These hybrid peptides were termed as FV-LL (FV7 followed by residues 17–29 of LL-37), FV-MA (FV7 followed by residues 9–21 of magainin 2) and FV-CE (FV7 followed by residues 1–8 of cecropin A) respectively (Table 1). The aim of this study was to investigate the effect of different types of hybrid peptides on their antibacterial and biological activities. 

FV-LL and FV-CE exhibited a higher potential to form an amphiphilic and stable structure than FV7 and the hybrid peptide FV-MA (Table 1 and Figure 1), although all the designed hybrid peptides were inherently amphiphilic and stable. Most natural amphiphilic AMPs were previously reported to adopt a flexible random structure in aqueous solutions, however, they assemble into an obvious α-helical structure in simulated-membrane environments [13]. The secondary structures of these peptides in both aqueous solution and membrane-mimetic environments were determined in this study. The results indicated that the hybrid peptides exhibited the same characteristic α-helical conformation, which was consistent with the predictions obtained through I-TASSER (Figure 1). Consequently, FV-LL, FV-MA and FV-CE can be defined as amphipathic α-helical peptides.

Compared to the parental peptide FV7, the three hybrid peptides, especially FV-LL, possessed remarkable antibacterial activities against Gram-negative strains and Gram-positive strains. Several factors, including α-helix propensity, positive charge and amphiphilicity, most likely caused these increases. In Table 1, FV-LL (+7) displayed a higher net charge than the hybrid peptide FV-MA (+5) and the parental peptide FV7 (+4), which was similar to FV-CE (+7). FV-LL also exhibited the highest antibacterial activity, which was consistent with previous reports that described an increase in cationic charge favoring the initial peptide electrostatic attractions to negatively charged bacteria membranes [31]. Meanwhile, the propensity for α-helix formation and amphiphilicity may play the most important roles in the design of hybrid peptides.

Based on the CD spectra, the parental peptide FV7 displayed an obvious β-sheet structure and all of the hybrid peptides displayed typical random coil structures in aqueous solution. In the anionic micellar environment (SDS), all three hybrid peptides displayed helical content, especially FV-LL, which showed a more obvious α-helical structure. The hybrid peptides showed their conformational change respectively in the different environments of SDS and TFE. During the research of CD spectra, neutral SDS was applied to observe the behavior of antimicrobial peptides as a membrane-mimicking environment [20]. It is reported that the structure of the α-helix peptide in TFE environment had a similar effect to that in SDS micelles at an acidic pH [32]. This conformational content was consistent with our hypothesis, which further confirmed the previous reports [33,34]. These data suggest that the α-helical structural characteristics of peptides may play a vital role in bacterial cell death, which may not prove to be correlated with antibacterial activity in Table 2.

Being less toxic against host cells is important for designed hybrid peptides as future biomaterials. The hemolytic assay showed that FV-LL had a lower hemolytic activity that was similar to the parental peptide FV7. However, the control natural peptide ME26 and FV-MA showed high hemolytic activities. Presumably, this may be because of hydrophobicity. Structural parameters such as higher hydrophobicity and helicity are correlated to improve hemolytic activity frequently [33,35], and the α-helical typical structure of FV-LL is likely to induce low hemolysis, presenting an improved selectivity towards the anionic component of bacterial cell membranes over zwitterionic mammalian cell membranes. This was presumably contributed to by the rational amphiphilicity of these peptides [36]. For *P. aeruginosa* PAO1, we found that the hybrid peptide FV-LL was able to inhibit biofilm formation and exhibit reasonable anti-biofilm activity compared to the parental peptide FV7. Indeed, FV-LL showed better inhibition performance than FV7. It has been reported that the capability of FV7 to inhibit biofilm had a connection with the decrease of bacteria cells reaching the surface [37].

According to previous reports, the addition of cations can affect the antibacterial activity of AMPs [38]. In this study, cations, especially Na^+^ and Mg^2+^, influenced the MIC values of peptides against Gram-negative and Gram-positive bacteria. After adding cations, the designed hybrid peptides, including FV-LL and FV-CE, showed similar antibacterial activities to not only Gram-negative bacteria but also Gram-positive bacteria. The bactericidal processing of cationic AMPs is generally rapid for both Gram-negative and Gram-positive bacteria, occurring in a stepwise manner [14]. It is known that the electrostatic forces between cationic peptides and bacteria membrane are the premise for peptides killing Gram-negative and Gram-positive bacteria [39,40]. AMP antibacterial activity may decrease because of the weakening of electrostatic interactions between peptides and bacteria. As a whole, the great antibacterial activity in the presence of salts and heat gives further promise to future prospects of this class of compounds toward new antibacterial agents (Table 3).

As the main target of most natural AMPs, outer membranes of bacterial cell, which are composed of hydroxylated phospholipids such as phosphatidylserine (PS), cardiolipin (CL) and phosphatidylglycerol (PG), have a predominantly negative net charge at physiological pH, and promote the effective binding of cationic AMPs [41,42]. After reorienting and inserting into the cytoplasmic membrane lipid bilayer, the peptide could lead to the disruption of membrane permeabilization and integrity or the formation to pore/ion channels, which could be concomitant with membrane electrical potential collapse [4,41]. Meanwhile, the results of this study indicated that hybrid peptides could disrupt the outer membrane rapidly which followed a concentration-dependent manner in response (Figure 4). Furthermore, all of the peptides displayed the ability to permeabilize the inner membrane to ONPG at 1× MIC concentration. The increase of cytoplasmic membrane potential caused by membrane disruption led to leakage of cell cytoplasmic content and cell death. Our SEM and TEM data further confirmed that all of the hybrid peptides displayed strong membrane disruption and intracellular contents leakage potency. FV-LL displayed similar membrane damage mechanism to a greater extent, which is consistent with its different microbicidal ability.

In conclusion, we designed a series of synthetic cationic hybrid antimicrobial peptides based on the peptide FV7 combined with fragments of the different natural peptides LL-37, magainin 2 and cecropin A, which have previously been determined to be optimal for antibacterial activity and cell selectivity. We aimed to investigate the benefits of hybrid peptides on antibacterial activity, selectivity and hemolytic activity. These advantageous properties were particularly due to the hybrid peptides serially combining different functional sequences to form α-helical conformations and amphiphilic structures. The hybrid peptides may have exerted their bactericidal effects by forming pores to damage the integrity of membranes, thereby causing cytosolic leakage and cell death. Additionally, the hybrid peptides, particularly FV-LL, demonstrated significant antibacterial activity against Gram-negative and Gram-positive bacteria and a lower degree of hemolysis. Based on the strengths and weaknesses of these hybrid peptides, this type of rational design will be useful for future assessments to develop and apply these AMPs as antibacterial agents. Our research supports the use of hybrid peptides as new-style biomaterials and the need for further studies aimed at evaluating the activity of peptides against infection in animal models.

## 4. Materials and Methods

### 4.1. Materials

Phosphate-buffered saline (PBS) solution obtained from Kermel (Tianjin, China), sodium dodecyl sulfate (SDS) obtained from Sigma-Aldrich (Shanghai, China) and trifluoroethanol (TFE) purchased from Amresco (Solon, OH, USA) were used for CD as dilution agents. Mueller-Hinton Agar (MHA) powder and Mueller-Hinton Broth (MHB) powder were obtained from AoBoX (Beijing, China) to incubate the bacteria. Ethanol, acetone, and tertiary butanol which were analytical grade (>99%) were all purchased from Sigma-Aldrich (Shanghai, China). Triton X-100, *N*-phenyl-1-naphthylamine (NPN), HEPES, and glutaraldehyde (synthetic grade, 50% in H_2_O) were obtained from Sigma-Aldrich (Shanghai, China).

### 4.2. Sequence Analysis of the Peptides

The primary peptide physicochemical parameters and sequences were calculated using bioinformatics programs including ProtParam (ExPASy Proteomics Server) [43] and the antimicrobial peptide database [44]. The secondary structure type of each residue was predicted online using Jpred 3 [45] and I-TASSER [46].

### 4.3. Synthesis of the Peptides

All of the peptides used in the study, including the parental peptide FV7, LL, MA, CE and the hybrid peptides FV-LL, FV-MA and FV-CE, were synthesized and purified by GL Biochem Corporation (Shanghai, China) through solid-phase methods using *N*-9-fluorenylmethyloxycarbonyl (Fmoc) chemistry. The purity was determined to be greater than 95% by reverse-phase high-performance liquid chromatography (RP-HPLC). The true molecular masses were further confirmed through matrix-assisted laser desorption/ionization time-of-flight mass spectroscopy (MALDI-TOF MS, Model Autoflex, Bruker Daltonics Inc., Billerica, MA, USA). The peptides were identified through electrospray mass spectrometry and dissolved in deionized water at a concentration of 2.56 mM. The peptide solutions were stored at −20 °C before the structural and bacteriostatic ability assessments.

### 4.4. Circular Dichroism (CD) Spectra

To investigate conformational changes induced by membrane environments, the hybrid peptides and parental peptides were measured at 25 °C by a CD spectropolarimeter (Jasco, J-820, Tokyo, Japan) equipped with a rectangular quartz cell with a 0.1 cm path length to the circular dichroism (CD) spectra. The spectra were recorded at wavelengths ranging from 190 to 250 nm at a 10 nm/min scanning speed. The solutions were prepared at a 150 μM peptide concentration in 10 mM of PBS (pH 7.4, to mimic an aqueous environment), 30 mM of SDS micelles (negatively charged prokaryotic membrane comparable environment) and 50% TFE (to mimic the hydrophobic environment of the microbial membrane). An average of at least three scans was conducted for each peptide sample. The acquired CD signal spectra were converted to the mean residue ellipticity according to the following equation:

θ_M_ = (θ_obs_·1000)/(c·l·n)

where θ_M_ is the mean residue ellipticity (deg·cm^2^·dmol^−1^), θ_obs_ is the observed ellipticity corrected for the buffer at a given wavelength (m·deg), c is the peptide concentration (mM), l is the path length (mm) and n is the number of amino acids. The percentage of α-helical structure was calculated as following:

$$\alpha -\mathrm{helical}\;\mathrm{content}\;\left(\%\right)=\left\{\frac{{\left[\theta \right]}_{222}-{\left[\theta \right]}_{0}}{{\left[\theta \right]}_{100}-{\left[\theta \right]}_{0}}\right\}\cdot 100$$

where ${\left[\theta \right]}_{222}$ is the experimentally observed mean residue ellipticity at 222 nm, and values for ${\left[\theta \right]}_{100}\;\mathrm{and}\;{\left[\theta \right]}_{0}$ which correspond to 0% and 100% α-helix content at 222 nm, are estimated to be −2000 and −32,000, respectively [27,37].

### 4.5. Antimicrobial Assays

Minimum inhibitory concentration (MIC) was determined using the modified standard microtiter dilution method as described previously. The bacteria were incubated overnight at 37 °C, centrifuged at 220 rpm and transferred to new nutrient broth (MHB) until the logarithmic phase of growth. The bacteria were then diluted into 10^5^ colony forming units (10^5^ CFU/mL) in MHB. The peptides were dissolved and diluted in 0.01% acetic acid and 0.2% bovine serum albumin (BSA). Briefly, serial diluted (0.5–128 µM) peptides were added to sterile 96-well plates in a 50-μL volume of bacteria in MHB mixed with 50 µL. After incubation at 37 °C for 18–24 h in an incubator, the MIC was determined as the lowest concentration of the peptide that resulted in no bacterial growth similar to the negative control with pure broth without microbes. The results are expressed as an average of the MICs at least three times. 

*E. coli* ATCC 25,922, *S. typhimurium* ATCC 7731, *S. typhimurium* ATCC 14028, *P. aeruginosa* ATCC 27853, *B. subtilis* CMCC 63501, *S. aureus* ATCC 29213, *S. epidermidis* ATCC 12228 and *E. faecalis* ATCC 29212 were obtained from the College of Veterinary Medicine, Northeast Agricultural University (Harbin, China). *E. coli* UB1005 was kindly provided by Qingsheng Qi (State Key Laboratory of Microbial Technology, Shandong University, Jinan, China). *P. Aeruginosa* PAO1 was kindly provided by Wengong Yu (State Key Laboratory of School of Medicine and Pharmacy, Ocean University of China, Qingdao, China).

### 4.6. Measurement of Hemolytic Activity

The hemolytic activity of the peptides was evaluated by measuring the amount of released hemoglobin by the lysis of erythrocytes. The measurement was as previously described [47]. Human red blood cells (hRBC) were obtained from healthy donors by the ethics committee of the Northeast Agricultural University Hospital. The fresh blood cells were washed 2–3 times in PBS (pH 7.4) and centrifuged at 1000× *g* for 5 min at 4 °C, then resuspended in PBS. Briefly, 50 μL of hRBC solution was incubated with 50 μL of different concentrations of each peptide dissolved in PBS in a 96-well microtiter plate. After incubation for 1 h at 37 °C, the mixtures were centrifuged at 1000× *g* for 5 min at 4 °C and then the supernatant was transferred to another 96-well microtiter plate. The release of hemoglobin was monitored by measurement of the absorbance at 570 nm using a multimode microplate reader (SpectraMax M5, Molecular Devices, San Francisco, CA, USA). The concentration causing 5% hemolysis was defined as the minimal hemolytic concentration (MHC). The control samples for 0% and 100% hemolysis consisted of hRBC in PBS (A_blank_) only and in 0.1% Triton X-100 (A_triton_), respectively. The percent hemolysis was calculated according to the following equation:

% Hemolysis = [(A_sample_ − A_blank_)/(A_triton_ − A_blank_)] × 100

### 4.7. Salt and Heat Sensitivity

To test the effect of different environments on the MIC assay of the peptides, bacteria containing both *E. coli* ATCC 25922 and *S. aureus* ATCC 29213 grown to the logarithmic phase in MHB were moderately diluted to 1 × 10^6^ CFU/mL and separately exposed to each peptide at 1× MIC in the presence of physiological salts at the following concentrations: (1) 150 mM NaCl; (2) 4.5 mM KCl; (3) 1 mM MgCl_2_; (4) 6 mM NH_4_Cl; (5) 8 mM ZnCl_2_; (6) 4 mM FeCl_3_ and (7) 2.5 mM CaCl_2_. The incubation to test for thermal stability occurred at 100 °C for 1 h. After these treatments, the procedures were the same as described above for the MIC assay [14].

### 4.8. Outer Membrane Permeability Assay

The outer membrane (OM) permeability activity against Gram-negative bacteria was determined by the uptake of *N*-phenyl-1-naphthylamine (NPN), a fluorescent dye that is sensitive to the outer membrane, as described previously [14,48]. Briefly, *E. coli* UB1005 grown to the logarithmic phase (OD600 = 0.4) at 37 °C in MHB were harvested by centrifugation at 1000× *g* for 10 min at 4 °C. The cells were washed twice with 5 mM of sodium HEPES buffer containing 5 mM of glucose, pH 7.4 and re-suspended to an OD600 of 0.2. NPN was added to 2 mL of the bacterial suspension to a final concentration of 10 mM. Aliquots of the peptides were added to the bacterial suspension to give varying final concentrations (1–8 μM). The background fluorescence was recorded (excitation λ = 350 nm and emission λ = 420 nm) using an F-4500 fluorescence spectrophotometer (Hitachi, Japan). The fluorescence was recorded as a function of time until there were no further increases.

### 4.9. Evaluation of the Inner Membrane Permeability

The inner membrane permeability activity against *E. coli* bacteria was determined by the uptake of ONPG. *E. coli* UB 1005 cells were cultured to mid-log phase in MHB medium containing 2% lactose at 37 °C, and harvested by centrifugation at 3000× *g* for 5 min. After washing twice, the cells were diluted to an OD600 of 0.05 with buffer (10 mM PBS, pH 7.4) containing 1.5 mM ONPG. The concentration of different peptides to 1× MIC was then incubated with bacteria cells. The value of OD reflected the ONPG flowing into the cells was measured at 420 nm from 0 to 30 min every two min.

### 4.10. Scanning Electron Microscopy (SEM) Characterization

For the SEM sample preparation, *E. coli* ATCC 25922 and *S. aureus* ATCC 29213 cells were grown to the logarithmic phase in MHB at 37 °C under constant shaking at 220 rpm, harvested by centrifugation at 1000× *g* for 10 min, washed twice with PBS and re-suspended to an OD600 of 0.2. Cells were incubated at 37 °C for 60 min with different peptides at their respective 1× MIC, with no peptide added as the control. After incubation, the cells were harvested by centrifuged at 5000× *g* for 5 min, washed 2–3 times with PBS, and fixed with 2.5% glutaraldehyde at 4 °C overnight, followed by washing with PBS. The cells were dehydrated for 10 min in a graded ethanol series (50%, 70%, 90% and 100%) and for 15 min in 100% ethanol, a mixture (*v*:*v* = 1:1) of 100% ethanol and tertiary butanol, and absolute tertiary butanol. The specimens were then dehydrated, dried with CO_2_, coated with gold-palladium and examined using a Hitachi S-4800 SEM (Hitachi, Tokyo, Japan).

### 4.11. Transmission Electron Microscopy (TEM) Characterization

Bacteria samples were initially prepared in the same protocol as for SEM. After pre-fixing with 2.5% glutaraldehyde at 4 °C overnight, bacterial cells were washed twice with PBS, post-fixed with 2% osmium tetroxide for 70 min, and washed twice with PBS (pH 7.2). The bacterial samples were dehydrated for 8–10 min in a graded ethanol series (50%, 70%, 90% and 100%), and for 10 min in 100% ethanol. Then, the specimens were transferred to mixtures (*v*:*v* = 1:1) of absolute acetone and epoxy resin for 30 min and then to pure epoxy resin and incubated overnight at a constant temperature. The specimens were sectioned using an ultramicrotome, and stained with uranyl acetate and lead citrate, and observed using a Hitachi H-7650 TEM (Hitachi, Tokyo, Japan).

### 4.12. Effect of Peptides on Biofilm Formation

A static biotic solid surface assay shows the capability of peptides to inhibit biofilm formation as described previously [19,49]. *P. aeruginosa* PAO1 bacteria were grown overnight in Tryptic Soy Broth (TSB). Then, the dilutions (1/100) of the colony were distributed into a 96-well plate with different concentrations of peptides. After incubation for 22 h at 37 °C, the wells were washed twice with PBS (pH 7.4) followed by the medium with planktonic cells was discarded. Crystal violet was used to strain the biofilm cells adhering to the side of tubes. After 20 min, the wells were rinsed with deionized (DI) water until the water was clear without visible purple color. The stained crystal violet was dissolved in ethyl alcohol and then measured at OD 570 nm with a microplate reader (Bio-Tek Instruments Inc., Winooski, VT, USA).

### 4.13. Statistical Analysis

All experiments were performed at least three times. All values were presented as the means ± standard error mean. Statistical comparisons were carried out by analysis of ANOVA with Social Sciences (SPSS) 16.0 software (IBM, Armon, NY, USA).

## 5. Conclusions

In this study, we designed a series of synthetic cationic hybrid antimicrobial peptides based on the peptide FV7 combined with fragments of different native peptides LL-37, magainin 2 and cecropin A, which have previously been determined to be optimal for antibacterial activity and cell selectivity, to investigate the benefits of hybrid peptides on antimicrobial activity, selectivity and hemolytic activity. These advantageous properties were particularly due to the hybrid peptides serially combining different functional sequences to form α-helical conformations and amphiphilic structures. The hybrid peptides may have exerted their bactericidal effects by forming pores to damage the integrity of membranes, thereby causing cytosolic leakage and cell death. Additionally, the hybrid peptides, particularly FV-LL, demonstrated significant antimicrobial activity against Gram-negative and Gram-positive bacteria and a lower degree of hemolysis compared to parental peptides. Based on the strengths and weaknesses of these hybrid peptides, this type of rational design will be useful for future assessments to develop and apply these AMPs as antibacterial agents. Our research supports the use of hybrid peptides as new-style biomaterials and the need for further studies aimed at evaluating the activity of peptides against infection in animal models.

## Figures and Tables

**Table 1:** Amino acid sequences, design, molecular weights, net charges and hydrophobicity values of the peptides used in this study.

Peptide	Sequence	Design ^1^	Theoretical Mw	Measured Mw ^2^	Charge	H ^3^
FV7	FRIRVRV-NH_2_	FV7	945.18	946.24	+4	0.57
LL	FKRIVQRIKDFLR-NH_2_	LL-37 (17–29)	1719.106	1718.14	+4	0.46
MA	AKKFGKAFVGEIM-NH_2_	magainin 2 (9–21)	1425.749	1424.78	+2	0.53
CE	KWKLFKKI-NH_2_	cecropin A (1–8)	1090.405	1089.45	+4	0.50
FV-LL	FRIRVRV-FKRIVQRIKDFLR-NH_2_	FV7 + LL-37 (17–29)	2646.27	2645.31	+7	0.50
FV-MA	FRIRVRV-AKKFGKAFVGEIM-NH_2_	FV7 + magainin 2 (9–21)	2352.92	2351.96	+5	0.55
FV-CE	FRIRVRV-AKKFGKAFVGEIM-NH_2_	FV7 + cecropin A (1–8)	2017.58	2016.62	+7	0.53

^1^ Design refers to the composition of hybrid peptides; ^2^ Molecular weight (MW) was measured by mass spectroscopy (MS); ^3^ The relative hydrophobic moment (μHrel) of a peptide is its hydrophobic moment relative to that of a perfectly amphiphilic peptide. This gives a better idea of the amphiphilicity using different scales. A value of 0.5 thus indicates that the peptide has ~50% of the maximum possible amphiphilicity.

**Table 2:** Antibacterial activities of the peptides.

	MIC ^1^ (μM)							
	FV7	LL	MA	CE	FV-LL	FV-MA	FV-CE	ME26
**Gram-negative bacteria**								
*E. coli* ATCC 25922	16	16	32	64	1	8	2	2
*E. coli* UB 1005	32	16	>64	>64	4	8	2	2
*P. aeruginosa* ATCC 27853	16	8	>64	>64	4	8	2	2
*P. aeruginosa* PAO1	32	16	>64	>64	2	2	2	2
*S. typhimurium* ATCC 14028	16	32	>64	>64	2	4	4	4
*S. typhimurium* ATCC 7731	32	16	>64	>64	2	4	4	4
**Gram-positive bacteria**								
*S. aureus* ATCC 29213	64	16	>64	>64	2	8	8	8
*S. faecalis* ATCC 29212	32	32	>64	>64	4	16	1	1
*B. subtilis* CMCC 63501	16	16	>64	>64	4	4	1	1
*S. epidermidis* ATCC12228	32	8	>64	>64	1	8	0.5	0.5

^1^ Minimum inhibitory concentrations (MIC) were determined as the lowest concentration of the peptides that inhibited bacteria growth. The tests were performed at least three times in duplicate.

**Table 3:** MIC values of peptides in the presence of physiological salts and heat with *E. coli* ATCC 25922 and *S. aureus* ATCC 29213.

Peptide	Control ^1^	NaCl ^2^	KCl ^2^	NH_4_Cl ^2^	MgCl_2_^2^	ZnCl_2_^2^	FeCl_3_^2^	CaCl_2_^2^	Mix ^3^	Heat (100 °C)
*E. coli* ATCC 25922										
FV7	16	32	64	>128	32	32	128	64	128	16
FV-LL	1	1	1	2	2	2	2	2	2	1
FV-MA	8	8	8	8	8	8	8	8	8	16
FV-CE	2	2	2	2	2	2	4	2	2	2
ME26	2	4	2	2	4	4	2	2	4	2
*S. aureus* ATCC 29213										
FV7	64	128	64	64	64	32	64	64	128	64
FV-LL	2	2	2	2	4	2	4	2	4	2
FV-MA	8	32	8	8	8	8	8	16	16	8
FV-CE	2	2	2	2	2	2	4	2	4	4
ME26	8	4	4	2	2	2	2	4	4	8

^1^ Minimum inhibitory concentrations (MICs) were texted as the lowest concentration of the peptides that inhibited bacteria growth; ^2^ The final concentrations of NaCl, KCl, NH_4_Cl, MgCl_2_, CaCl_2_, ZnCl_2_, and FeCl_3_ were 150, 4.5, 6, 1, 2.5, 8, and 4 mM, respectively, and the control MIC values were determined in the absence of these physiological salts; ^3^ The mixture medium contained all kinds of salts in physiological concentrations.
